# Colorectal Cancer Characteristics and Outcomes after Solid Organ Transplantation

**DOI:** 10.1155/2019/5796108

**Published:** 2019-02-28

**Authors:** Amit Merchea, Faisal Shahjehan, Kristopher P. Croome, Jordan J. Cochuyt, Zhuo Li, Dorin T. Colibaseanu, Pashtoon Murtaza Kasi

**Affiliations:** ^1^Division of Colon & Rectal Surgery, Mayo Clinic, Jacksonville, FL, USA; ^2^Division of Hematology and Oncology, Mayo Clinic, Jacksonville, FL, USA; ^3^Division of Biomedical Statistics & Informatics, Mayo Clinic, Jacksonville, FL, USA; ^4^Division of Hematology and Oncology, University of Iowa, Iowa City, IA, USA

## Abstract

**Background:**

Individuals after solid organ transplant may develop secondary malignancies. In our clinical practice, we noted an increasing number of individuals who developed colorectal cancers after solid organ transplantation. The primary aim of this study was to describe the characteristics and outcomes of the patients who developed colorectal cancer after solid organ transplant.

**Materials and Methods:**

Data was gathered and merged from several registries at Mayo Clinic to identify all patients who received a diagnosis of colon or rectal cancer and solid organ transplant. Continuous variables were summarized as mean (standard deviation) and median (range), while categorical variables were reported as frequency (percentage). Time to colorectal cancer after transplant and overall survival after cancer diagnosis were estimated using Kaplan-Meier method.

**Results:**

Initially, 115 colorectal cancer patients who also had a transplant were identified. The diagnosis of colorectal cancer was noted after solid organ transplant in 63 patients. The mean age at transplant was 57 years. Majority had received a kidney transplant (44.4%) followed by liver (36.5%). The median time to develop colorectal cancer was 59.3 months (range: 4.4-251.4 months). 15 (24.6%) were stage 4 at diagnosis and 13 (21.3%) had stage 3 colorectal cancer. Median overall survival was 30.8 months; 5-, 10- and 15-year survival were noted to be 42.5%, 17.9%, and 7.5%, respectively. None of the stage 4 patients were alive at 5 years; 5-year survival rate for stage 1, 2, and 3 patients was 77%, 50%, and 42%, respectively.

**Conclusions:**

Our study reports on one of the largest cohorts of patients of colorectal cancer that developed the cancer after solid organ transplant. Survival is extremely poor for advanced cases. However, long-term survivors are noted who developed the cancer at a relatively early stage. Colorectal screening recommendations may need to be revised for patients after solid organ transplant.

## 1. Introduction

Patients after organ transplant are at an increased risk of developing secondary malignancies. Cancer is considered as the third leading cause of death in solid organ transplant patients after cardiovascular events and infections [1]. The development of cancer in posttransplant patients was first reported in 1960s [2, 3]. Reported incidence patterns of cancer in this patient population are variable, but reports have shown an overall increased risk of cancers in patients after solid organ transplant as compared to general population [4, 5].

Over the last 2 years, we saw several patients at our center who were diagnosed with colorectal cancer secondary to transplant-related immunosuppression. Given the paucity of data in the literature, we wished to evaluate the outcomes and characteristics of colorectal cancer (CRC) in solid organ transplant recipients [6]. We, therefore, decided to investigate this further since CRC has been reported in individuals who had solid organ transplantation, although the data is limited [7].

The specific objectives of this study were (1) to describe the patient population who developed CRC after solid organ transplantation at our institution, and (2) to review the literature about the development of CRC after solid organ transplantation.

## 2. Materials and Methods

Institutional review board approval was obtained. A retrospective observational study utilizing the Mayo Clinic Cancer Center (Minnesota, Florida, and Arizona) Registry was performed to identify solid organ transplant (heart, kidney, liver, lung, and pancreas) patients who were later diagnosed with CRC from 1987-2016. Patients with a diagnosis of CRC preceding their transplant were excluded. Data on demographics, tumor characteristics, and survival was gathered by reviewing the records and registry date.

Continuous variables were summarized as mean (standard deviation) and median (range), while categorical variables were reported as frequency (percentage). Time to CRC after transplant and overall survival after cancer diagnosis were estimated using Kaplan-Meier method.

## 3. Results

The study population included 63 patients who developed CRC after solid organ transplantation. We initially identified 115 patients who had CRC as well as organ transplantation, but our data showed that only 63 patients received transplants before the CRC diagnosis.

The mean age of our patient cohort at time of transplant was 57.3 ±9.6 years (range: 27.3-75.5) and at the time of CRC diagnosis was 63.4 ±8.2 years (range: 37.0-82.0). More than half of patients were male (55.6%). The most common transplanted organs in the cohort were kidney (44.4%) and liver (36.5%) followed by heart (11.1%) and lung (6.3%).

**Figure 1:**
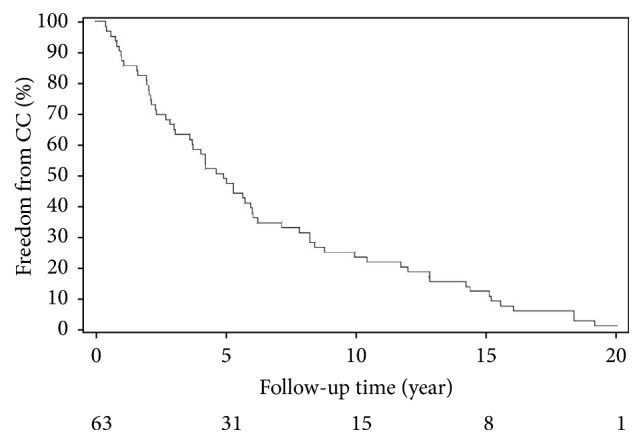
Time to colorectal cancer after organ transplant.

The proportions of patients who remained cancer free at 5, 10, and 15 years after transplant were 49.2% (95%CI: 38.3%-63.2%), 23.8% (95%CI: 15.3%-37.0%), and 12.7% (95%CI: 6.7%-24.3%), respectively. The median time to develop CRC was 59.3 months (range: 4.4-251.4 months). Time to CRC after organ transplant is demonstrated in Figure 1. Demographic and baseline variables at cancer diagnosis are described in Table 1.

The majority of posttransplant patients developed tumor of the right colon (60.9%) as compared to the left colon (23.9%) and rectum (15.2%) (transverse colon and rectosigmoid colon excluded from this classification). Of the 63 transplant recipients, 13 (21.3%) had stage 3 disease and 15 (24.6%) patients had stage 4 disease. Cancer related information is presented in Table 2.

**Figure 2:**
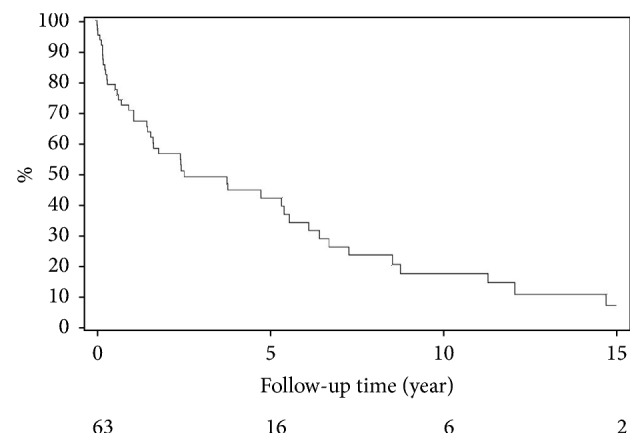
Overall survival after colorectal cancer diagnosis.

**Figure 3:**
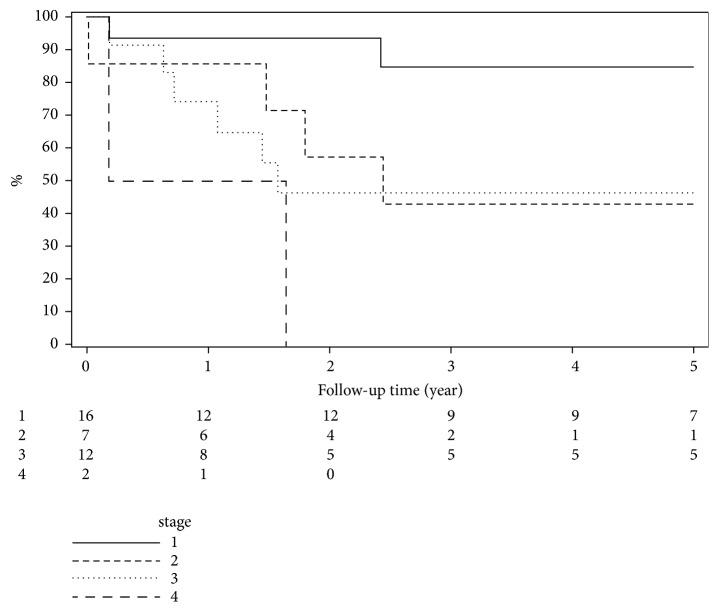
Survival after colorectal cancer diagnosis by stage.

The 5-, 10-, and 15-year overall survival of posttransplant CRC patients were estimated to be 42.5%, 18%, and 7.5%, respectively. The median overall survival was 30.8 months. Overall survival and stage-specific survival since cancer diagnosis were calculated by Kaplan-Meier method and demonstrated in Table 3 and Figures 2 and 3, respectively. Five years after diagnosis, the stage-specific survival was 77% for stage 1, 50% for stage 2, 42.3% for stage 3, and 0.0% for stage 4 patients.

## 4. Discussion

This study of our 3-site, single institutional Cancer Registry of transplant patients developing colorectal cancer found the following: (1) on average patients developed CRC 6.1 years after transplant, (2) majority of cancers were right-sided, (3) a significant percentage of patients (24.6%) presented with stage IV disease, and (4) 5-year overall survival was favorable when diagnosed early (overall 5-year survival was noted to be 42.5%). To our knowledge, this is one of the largest posttransplant CRC patient data ever reported.

Previous studies have demonstrated the association between solid organ transplantation and development of cancer; among those, most of the patients had hematologic and skin cancers [5, 8, 9]. Summary of the studies showing the development of cancer after receiving solid organ transplant is demonstrated in Table 4. We specifically studied development of CRC in solid organ transplant patient population. Previously, a study by our group at Mayo Clinic demonstrated the development of CRC in 20 patients out of 3,946 transplant recipients [6]. Similarly, Rocha et al. reported data on 60 patients who developed noncutaneous cancers in a cohort of 1695 (3.5%) patients after kidney transplantation. Of these, 21.2% of the noncutaneous cancers were CRC, followed by lymphoma and breast cancer [1].

Our study described the patient and cancer characteristics of a cohort of patients diagnosed with CRC at a single institution after receiving solid organ transplant and determined their outcomes in terms of survival in these patients. The majority of CRC patients had kidney and liver transplants in this study. The results of several other studies have also shown the development of cancer of any type after receiving kidney or liver transplant [9–13]. However, few studies specifically showed the development CRC after having kidney or liver transplant [1, 7]. A meta-analysis of 29 studies showed an increased risk of CRC in liver transplant patients compared to general population (overall RR=2.6, 95% CI: 1.7-4.1) [14]. Recently, Kang et al. studied 17 CRC cases after receiving liver transplant and reported an increased risk of CRC development in posttransplant population with an estimated odds ratio of 3.6 (p=0.001) [15].

Little data exists regarding the etiology, pathogenesis, and underlying factors that may impact CRC after solid organ transplantation. However, some factors have been identified that might have a role. Some patients, for example, who get a liver transplant secondary for primary sclerosing cholangitis, have underlying ulcerative colitis to begin with, which is associated with an increased risk for development of CRC regardless of the transplant [16–18]. Carenco et al. studied 465 liver transplant patients for the development of cancer and reported pretransplant smoking (odds ratio=5.5, 95% CI: 2.5-12; P < 0.0001] and obesity (odds ratio=2.2, 95% CI: 1.1-4.3; P = 0.0184) as two factors increasing the risk of cancer in posttransplant patient population [13]. Another study reported alcoholism as an independent risk factor for occurrence of cancer after receiving solid organ transplantation [19]. Whether having history of treated pretransplant cancer is a risk factor for cancer recurrence after solid organ transplant is not well established. However, a study conducted on 30 patients having cancer before transplant reported a recurrence rate of 3% after receiving transplant [20]. Several viruses have been found to be associated with the development of cancer in posttransplant population because of immunosuppression. Epstein-Barr virus is one of the most notable and established pathogens which is associated with development of posttransplant lymphoproliferative disorder [21]. According to few studies, cytomegalovirus and hepatitis C virus may also play a role in development of cancer in posttransplant population [21, 22]. It has been reported that the overall incidence rate of post-liver transplantation lymphoproliferative disorder is 1-4% [23, 24].

It is also intriguing to note that majority of the colon cancers that developed were on the right side. We now know that the biology and origin of left- and right-sided cancers are different, and the immunosuppression may have a role to play [15]. However, these findings are only hypothesis generating at this point given the single institution and retrospective nature of our study.

We also determined overall survival of posttransplant CRC patients and further stratified the patients to estimate stage-specific survival. The results indicate that survival decreases as both the time from diagnosis and disease stage increases, with zero percent survival for stage 4 patients at 5 years after diagnosis. In our study, 24.6% patients were diagnosed at tumor stage 4. In another study from Hungary, 43.5% posttransplant CRC patients were diagnosed at tumor stage 4 and 5-year survival rate was calculated to be 13.9% [25]. Merchea et al. studied 20 posttransplant CRC patients and reported the proportion of patients diagnosed at stage 4 as 30% and 5-year overall survival for all stages as 69% [6]. Papaconstantinou et al. examined data of 150 posttransplant CRC patients from two registries and reported that CRC patients after receiving solid organ transplants had worse 5-year overall survival compared to general population (44% vs. 62%, p<0.001) [26]. An Asian study also showed that there is poor survival in CRC patients diagnosed after kidney transplant compared to general CRC patient population and reported 2-year survival rate for advanced CRC in transplant group (45.7%) and nontransplant group (71.6%) [27]. The poor survival outcomes in these patients is a cause for great concern since they are getting a transplant as curative-intent and then to be dying from a secondary cancer, which can be potentially prevented or screened for is worrisome.

Furthermore, several studies have shown that solid organ transplant recipients in European population are at an increased risk of developing cancer compared to general population. Adami et al. studied 5931 solid organ transplant patients from Swedish population and reported an increased risk of cancer in solid organ transplant recipients with an estimated overall standardized incidence ratio (SIR), SIR for colon cancer, and SIR for rectal cancer of 4.0 (95% CI: 3.7-4.4), 2.3 (95% CI: 1.5-3.4), and 1.9 (95% CI: 1.0-3.2), respectively [9]. Haagsma et al. studied 174 liver transplant patients in Netherlands and reported the overall risk of having cancer after transplant at 5, 10, and 15 years as 6%, 20%, and 55%, respectively [10]. The said study also reported relative risk (RR) for colon cancer (RR=12.5, 95% CI: 2.5-36.6) but only three patients, out of total 21 who got cancer in this study, developed colon cancer [10]. Sint Nicolaas et al. studied 394 liver transplant patients in Netherlands and estimated an overall SIR of 2.16 (95% CI: 0.81-5.76) for CRC compared to general population [7]. Kyllönen et al. did a study in Finland on kidney transplant recipients and reported an increased risk of cancer in this population with calculated overall SIR=3.33 and SIR for colon cancer equal to 3.9 [11]. Brunner et al. analyzed data of several registries from England, Wales, Sweden, Germany, and Italy and reported the similar results of increased risk of cancer following kidney transplantation [12]. Aigner et al. studied 3595 solid organ transplant recipients in Austria and reported that 206 (5.7%) developed cancer among whom 9 got CRC (0.25%) [28]. These abovementioned studies well established the association of increased risk of cancer and solid organ transplantation in European population.

Similar findings were stated by studies done in the United States. Albright et al. conducted an observational study on 402 liver transplant patients and found that 3 patients developed CRC [19]. Engels et al. examined data of 175,732 solid organ transplant recipients from several US registries and reported an increased overall cancer risk in posttransplant population with an estimated SIR of 2.10 (95% CI: 2.06-2.14) [29].

Contrary to the results of the aforementioned studies, a recent study showed a decreasing trend in incidence of cancer in liver transplant recipients over the last three decades and calculated SIRs for 1980s, 1990s, and 2000s as 4.53, 3.17, and 1.76, respectively [16].

The limitations of our study include retrospective nature of the study, the relatively small sample size, and lack of a control group. Furthermore, there is lack of information regarding immunosuppression and therapy received.

## 5. Conclusion

In summary, we described the characteristics and outcomes of CRC patients who were diagnosed at our institution after receiving solid organ transplantation. We also reviewed the literature to understand and summarize the existing knowledge about the association between solid organ transplantation and risk of cancer. Our study reports on one of the largest cohorts of CRC patients that developed the cancer after solid organ transplantation. Survival is extremely poor for advanced cases. However, long-term survivors are noted who developed the cancer at a relatively early stage. CRC screening recommendations may need to be revised after solid organ transplant since individuals getting a transplant are getting it for curative-intent.

## Figures and Tables

**Table 1:** Baseline and demographic variables of patients who developed colorectal cancers after solid organ transplant.

	Total
	(N=63)

*Age at diagnosis*	
N	63
Mean (SD)	63.4 (8.2)
Median	64.0
Q1, Q3	58.0, 69.0
Range	(37.0-82.0)
*Age at diagnosis*	
<50	4 (6.3%)
50-60	16 (25.4%)
60-70	33 (52.4%)
70-80	9 (14.3%)
>80	1 (1.6%)
*Year of diagnosis*	
N	63
Mean (SD)	2006.3 (7.7)
Median	2008.0
Q1, Q3	2001.0, 2012.0
Range	(1987.0-2016.0)
*Year of diagnosis*	
1980-2000	13 (20.6%)
>=2000	50 (79.4%)
*Gender*	
Female	28 (44.4%)
Male	35 (55.6%)
*Race*	
White	59 (93.7%)
Black	1 (1.6%)
Asian/Pacific islander	2 (3.2%)
Other	1 (1.6%)
*Type of transplanted organ*	
Kidney	44.4%
Liver	36.5%
Heart	11.1%
Lung	6.3%
*Hospital site*	
Arizona	10 (15.9%)
Florida	8 (12.7%)
Rochester	45 (71.4%)

**Table 2:** Cancer-related information.

	Total
	(N=63)

*Cancer side (transverse colon excluded)*	
Missing	14
Right	28 (57.1%)
Left	21 (42.9%)
*Cancer location (transverse colon and rectosigmoid excluded)*	
Missing	17
Right	28 (60.9%)
Left	11 (23.9%)
Rectum	7 (15.2%)
*Tumor size*	
N	47
Mean (SD)	200.0 (361.6)
Median	35.0
Q1, Q3	23.0, 90.0
Range	(2.0-988.0)
*Regional lymph node positive*	
N	38
Mean (SD)	0.9 (1.6)
Median	0.0
Q1, Q3	0.0, 1.0
Range	(0.0-6.0)
*Regional lymph node exam*	
N	54
Mean (SD)	12.6 (15.6)
Median	8.5
Q1, Q3	0.0, 19.0
Range	(0.0-77.0)
*Stage*	
Missing	2
0	3 (4.9%)
1	20 (32.8%)
2	10 (16.4%)
3	13 (21.3%)
4	15 (24.6%)
*Follow-up years since cancer diagnosis*	
N	63
Mean (SD)	3.7 (4.7)
Median	2.3
Q1,Q3	0.6, 5.3

**Table 3:** Kaplan-Meier estimates of overall survival since cancer diagnosis.

Variable	# of	# of	5-yr survival	10-yr survival	15-yr survival	Median	Median
	Patients	Events	(95%CI)	(95%CI)	(95%CI)	Survival	Survival
						Time	Range
						(Months)	(Months)

*All*	63	46	42.47% (31.09,	17.92%	7.46%	30.8	(0.3, 292.17)
*patients*			58.01)	(31.09, 58.01)	(2.16, 25.74)		

*Stage 1*	20	11	77.09% (59.54,	25.70%	12.85%	81.67	(2.23,
			99.80)	(8.64, 76.39)	(2.20, 74.89)		292.17)

*Stage 2*	10	8	50.00% (26.90,	33.33%	N/A	29.73	(0.3, 178.97)
			92.93)	(12.11, 91.71)			

*Stage 3*	13	10	42.31% (21.83,	16.92%	16.92%	29.57	(2.3, 212.4)
			82.00)	(4.80, 59.73)	(4.80, 59.73)		

*Stage 4*	15	14	N/A	N/A	N/A	3.33	(0.43, 45.8)

**Table 4:** Summary of studies looking at solid-organ transplant and other cancers including colorectal cancers.

Author's name	Year published	No. of transplant patients in study	Transplanted organ(s)	Cancer(s) developed	Registry
Merchea et al. [6]	2014	20	(i) Kidney	CRC	Mayo Clinic
			(ii) Liver		Cancer Registry
			(iii) Heart		
			(iv) Lung		

Rocha et al. [1]	2013	1695	Kidney	(i) CRC (21.2%)	Single center,
				(ii) Malignant	Portugal
				Lymphoma	
				(16.7%)	
				(iii) Breast cancer	
				(13.6%)	

Albright et al. [19]	2010	402	Liver	(i) CRC (n=3)	Single center,
				(ii) HPV-anal	California USA
				cancer (n=2)	
				(iii) EBV-cecal	
				lymphoproliferative	
				cancer	
				(n=1)	

Carenco et al. [13]	2015	465	Liver	(i) Skin cancer	Single center,
				(n=28)	France
				(ii) PTLD (n=13)	
				(iii) Oral cancer	
				(n=17)	
				(iv) Lung cancer	
				(n=15)	
				(v) CRC (n=8)	
				(vi) Esophageal	
				cancer (n=4)	
				(vii) Anal cancer	
				(n=2)	
				(viii) Pancreatic	
				cancer (n=1)	

Aigner et al. [28]	2007	3595	(i) Kidney	(i) CRC (n=9,	Single center,
			(ii) Liver	0.25%)	Austria
			(iii) Pancreas	(ii) Anal cancer	
			(iv) Heart	(n=4, 0.11%)	
			(v) Lung	(iii) PTLD (n=5)	
			(vi) Small bowel		

Papaconstantinou	2004	150	(i) Kidney	CRC	Israel Penn
et al. [26]			(ii) Heart		International
			(iii) Liver		Transplant
			(iv) Lung		Tumor
					Registry, and
					National
					Cancer
					Institute SEER

Sint Nicolaas et al. [7]	2010	394	Liver	CRC (n=4, 1%)	Single center,
					Netherlands

Engels et al. [29]	2011	175,732	(i) Kidney (58.4%)	Non-Hodgkin	US Scientific
			(ii) Liver (21.6%)	lymphoma, Hodgkin	Registry of
			(iii) Heart (10.0%)	lymphoma, cancers of	Transplant
			(iv) Lung (4.0%)	stomach, liver, colon,	Recipients
				rectum, anus, oral	(1987-2008),
				cavity, vulva, cervix,	and 13 state
				penis, nasopharynx,	and regional
				vagina and others	cancer
					registries

Adami et al. [9]	2003	5931	(i) Kidney	Non-Hodgkin	National
			(ii) Liver	lymphoma, cancers of	cancer
				skin, lips, oral cavity,	database,
				esophagus, stomach,	Sweden
				colon, rectum, anus,	
				vulva, vagina, lung,	
				urinary bladder,	
				thyroid cancer	

Haagsma et al. [10]	2001	174	Liver	Cancers of skin, lip,	Single center,
				colon, kidney and B-	Netherlands
				cell lymphoma	

Kyllönen et al. [11]	2000	2890	Kidney	Cancers of skin, lip,	National
				thyroid, kidney, lower	transplant and
				urinary tract, colon,	cancer
				ovary and Non-	registries,
				Hodgkin lymphoma	Finland

Kang et al. [15]	2018	348	Liver	CRC	Single center,
					Korea

Nordin et al. [16]	2018	4246	Liver	CRC, Non-Hodgkin lymphoma, skin cancer	Nordic liver transplant registry

## Data Availability

The data used to support the findings of this study are available from Mayo Clinic Cancer Registry upon request.
